# Functional and Spatial Design of Emergency Departments Using Quality Function Deployment

**DOI:** 10.1155/2018/9281396

**Published:** 2018-11-14

**Authors:** Yassin Abdelsamad, Muhammad Rushdi, Bassel Tawfik

**Affiliations:** Department of Biomedical Engineering and Systems, Faculty of Engineering, Cairo University, Giza 12613, Egypt

## Abstract

Inadequate design of emergency departments (EDs) is a major cause of crowding, increased length of stay, and higher mortality. The main reason behind this inadequacy is the lack of stakeholders' involvement in the design process. This work reports and analyzes the results of a large survey of the requirements of ED stakeholders. It then compares these requirements with existing designs on the one hand and international standards on the other. Further, we propose a new hybrid design which combines the requirements of both the stakeholders and international standards using quality function deployment (QFD), also known as the House of Quality, method. The proposed method was used to assess two existing EDs located in two countries. The analysis of the survey responses showed certain discrepancies between stakeholder requirements and the existing designs such as the absence of an initial admission unit and insufficient space of the treatment unit. The results showed a strong correlation between the QFD-based design and stakeholder requirements (*r* = 0.92 for ED1 and *r* = 0.93 for ED2) which is attributed to the incorporation of stakeholders' opinions into the QFD method. The new design was also positively correlated to the international standards (*r* = 0.94 for ED1 and *r* = 0.91 for ED2). Our findings suggest that international design standards should be based on more structured methods for incorporating stakeholders' views and that a certain degree of difference should be allowed depending on the region in which the hospital is located to reflect both cultural and environmental differences.

## 1. Introduction and Background

The emergency department (ED) plays an important role in providing patients with prompt and effective clinical care [1]. It is the healthcare entry point responsible for receiving, sorting, assessing, stabilizing, and managing patients arriving at its door with different degrees of urgency and complexity. Conditions of patients requiring an emergency care vary from major trauma and stroke to intoxication and mental disorders [2, 3]. Therefore, ED is considered to be an extremely complex system [4]. Yet, its design has witnessed little, even if no progress in recent years, to cope with these complexities efficiently and cost-effectively.

Improper facility design can lead to numerous problems. In the ED in particular, crowding is a prominent problem [5–7]. As addressed by Carter et al. [8], crowding is associated with increased patient mortality, poor quality of care, extended waiting times, increased rates of patients left without being seen (LWBS), and extended length of stay (LOS). In addition to crowding, many healthcare professionals find current EDs incapable of meeting the expectations of patients, their families, and the medical staff.

To alleviate these ED problems, Welch [9] listed a number of factors that could improve the quality of ED service. Some of these factors involved human resources (such as the incorporation of medical teams), administrational controls (such as creating express admission units), and architectural design issues (such as the creation of intake pods zones, and discharge kiosks). Chan et al. [10] applied lean methodologies to improve the ED design.

Kolb et al. [11] proposed a five-buffer system to relieve pressure in the ED. This system reduced the triage-to-bed time by up to 22%, decreased the diversion by up to 24%, increased patient satisfaction, and improved the process flow. Tawfik et al. [12] used computerized relationship layout planning (CORELAP) to construct ED designs and create a new layout that includes major ED spaces. Results showed a reduction in patient crowding and an improvement in patient flow.

Although some of these studies involved stakeholders' opinions, such involvement was not integrated into the design process in a systematic and ordered way. One of the most popular methods known for involving stakeholder requirements is the quality function deployment (QFD) method, also known as the House of Quality (HoQ) [13, 14]. This method, developed by Toyota in the early Sixties, translates the customer requirements into appropriate technical specifications. It is one of the total quality management (TQM) tools [15]. The ultimate goal of QFD is to translate the subjective design criteria into objective ones to complete the design process in a systematic manner. Implementation of QFD takes four steps or phases, namely, (1) product planning, (2) parts design, (3) process planning, and (4) production planning [16].

**Figure 1:**
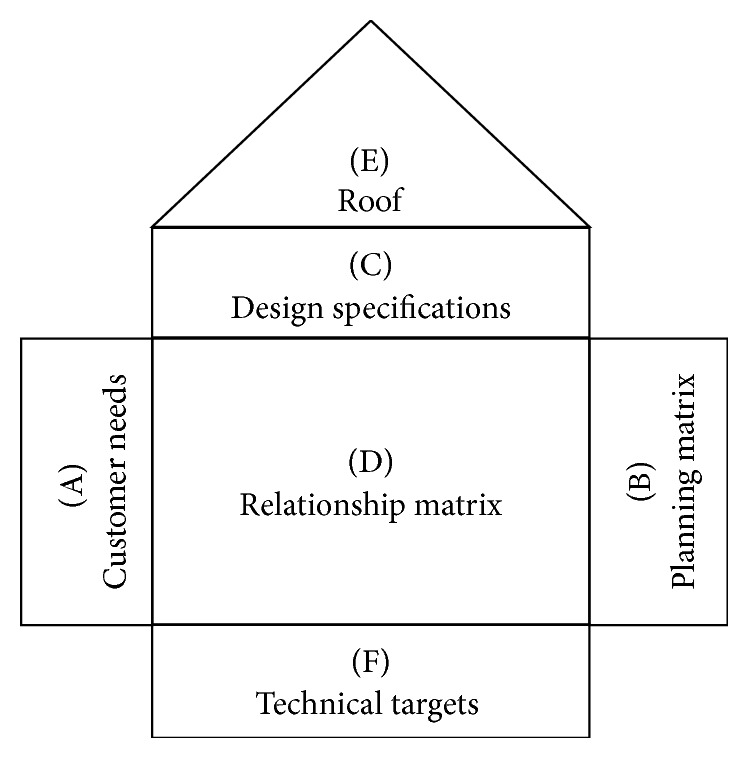
The House of Quality.

The House of Quality (HoQ) is a widely used form of QFD which consists of six rooms (Figure 1). The first room “A” contains the customer requirements. The assessment of customer requirements against business competitors takes place in room “B”. Room “C” has the technical characteristics which are a translation of customer needs, whereas room “D” includes the values of correlation between each customer requirement and technical specifications. The roof “E” shows how the design specifications support each other. The calculation of priorities of technical targets is done in room “F” [15, 17].

The HoQ has been traditionally used in the manufacturing business with the aim of systematically incorporating stakeholders' needs in future designs. Since the design process is fundamentally the same whether it is a machine or a facility, the HoQ methodology was extended to enhance the architectural design of a new children's nursery and in the design phase of an apartment construction project [17–19].

The objective of this paper is twofold. Firstly, we seek to take the quality function deployment (QFD) methodology one step further and apply it to propose a stakeholder-based design for the healthcare facilities. Secondly, we demonstrate the outcomes of the QFD methodology in the ED design. We have started with the ED not only because of its importance but also for its complexity which makes it a challenging quest. Towards this objective, we have collected the requirements of a large sample of ED stakeholders in two countries in the Middle East. We have then implemented the HoQ methodology in order to obtain an improved design over the existing ones. In doing so, we have made use of some of the most commonly used international standards (IS).

## 2. Materials and Methods

**Figure 2:**
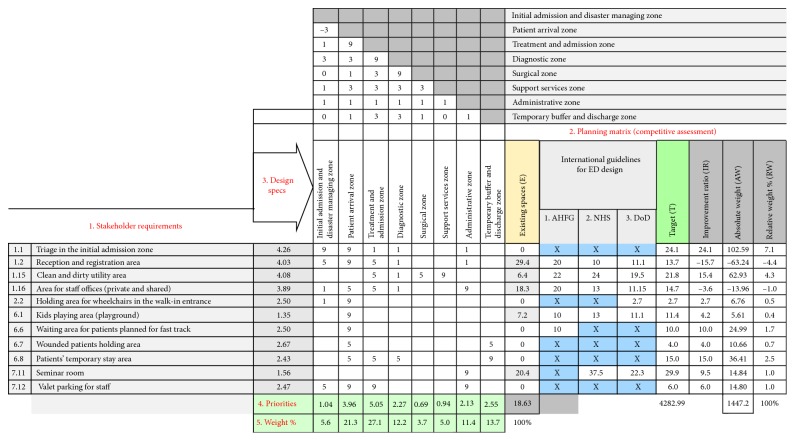
Part of the HoQ matrix for the QFD-based emergency department design.

As shown in Figure 2, the QFD-based ED design process seeks to identify and prioritize the ED functional units based on both stakeholder requirements (SR) and international standards (IS). Other design aspects such as environmental considerations, operational models, and architectural requirements are outside the scope of this work.

The new QFD-based design is obtained through the following steps.

### 2.1. Step 1: Listing Stakeholder Requirements

First of all, a focus group of ED stakeholders in Egypt and Saudi Arabia, including patients, was asked to mention those needs that they believe are important to improve the ED design, efficiency, and patient satisfaction. In addition, other concepts on ED design were collected from the guidelines of the Australasian College for Emergency Medicine [1], the Australasian Health Facility Guidelines [2], ED patient flow guidelines ([5, 9]), and ED overcrowding reduction concepts [11]. These concepts were used along with the inputs of the focus group to build a questionnaire of 84 items.

Then, we classified the survey items into eight groups, namely, (1) basic services, (2) requirements to streamline the material flow, (3) requirements to streamline the patient flow, (4) clinical support services, (5) consultation services, (6) areas to support the diagnostic services, (7) patient holding areas, and (8) areas for staff support and teaching.

The survey was then conducted for two months among 118 ED stakeholders, all of whom at the time of the survey were working in either Egypt or Saudi Arabia. The stakeholders can be roughly divided into two main groups. The first one includes strongly-affiliated ED staff (e.g., physicians, nurses, administrators, and technicians), whereas the second group includes loosely-affiliated professionals who do not work inside the ED but are linked to it (e.g., clinical engineers, healthcare planners, pharmacists, and non-ED physicians).

Out of 118 subjects, 108 completed the questionnaire with a response rate of 91.5%. The respondents were asked to rate the questionnaire items based on a 5-point importance scale (0 = not important, 1 = slightly important, 2 = moderately important, 3 = important, and 4 = extremely important). Then, the average importance value of each item was calculated and converted into a percent score.

### 2.2. Step 2: Development of the Design Specifications

This step is to translate the SR into relevant design specifications (DS) based on (1) brainstorming among experienced healthcare planners, (2) international guidelines, and (3) review of the literature.  Table 1 shows the proposed design specifications with their functions.

### 2.3. Step 3: Construction of the Relationship Matrix

The relationship matrix is the core of the QFD methodology. This matrix maps the correlation between SR and DS. According to Franceschini et al. [20], a 3-point ordinal scale (“weak”, “medium,” and “strong”) establishes the values of the relationship matrix using two series (0, 1, 3, and 9 or 0, 1, 3, and 5). The first series is derived from a logarithmic interval scale with 3 as a basis, while the second is derived from a linear interval scale. If the ratings derived from a linear interval scale are interpreted as being derived from a proportional scale, this can lead to a wrong priority rank of design specifications [20]. In this QFD-based method, we used the linear interval scale as (1 = low, 5 = medium, and 9 = high).

A correlation value reflects how a design specification supports the purpose of a particular SR. For example, as shown in Figure 2, the wounded patients who do not need additional treatment in the arrival unit (fast track) can be managed by allocating a temporary buffer and discharge unit to hold those patients until discharge.

### 2.4. Step 4: Construction of the Correlation Matrix (Roof)

This matrix shows how the design specifications support each other to identify the bottlenecks and trade-off. The six-point scale has been used in this study in which +9 represents strong positive correlation while -9 represents a strong negative correlation. For example, the correlation between (the Initial Admission and Disaster Management Unit) and (the Diagnostic Zone) is +3. This means that if the space of the first zone increased, the space of the second zone should be fairly increased as well.

### 2.5. Step 5: Development of the Planning Matrix

In the QFD literature [17, 21], the planning matrix includes the assessment of features of existing products against those of competitors. We modified this matrix to make the QFD methodolgy more suitable for healthcare facility design. The modified matrix includes the spaces of each service according to the existing design, SR, and recommendation of three IS, namely, Australasian Health Facility Guidelines (AusHFG) [2], National Health Service (NHS) [22], and United States Department of Defense (DoD) [23]. Here are the steps to create the planning matrix:

(1) Calculate the average importance *I*_*i*_ of each requirement from the questionnaire responses
(2) Calculate the existing space *E*_*i*_ of each requirement using the schematic diagram
(3) Find the space (*S*_*is*_) of each requirement according to (AusHFG, NHS, and DoD)
(4) Compare *E*_*i*_ with *S*_*is*_ for each requirement
(5) Set the target area *T*_*i*_ for each requirement as the average value of the three IS
(6) Calculate the improvement ratio *IR*_*i*_ as
  (1) $$\begin{array}{c} IR_{i}=T_{i}-E_{i}, \end{array}$$

where *T*_*i*_ and *E*_*i*_ are the target and the existing spaces for the *i*th requirement

(7) Calculate the absolute weight *AW*_*i*_ as
  (2) $$\begin{array}{c} AW_{i}=IR_{i}\;*\;I_{i}, \end{array}$$

where *IR*_*i*_, and *I*_*i*_ are the improvement ratio and importance score of the *i*th requirement, respectively

(8) Calculate the relative weight *RW*_*i*_ as
  (3) $$\begin{array}{c} RW_{i}=\frac{AW_{i}}{\sum_{i=1}^{n}AW_{i}}*100, \end{array}$$

where *n* is the number of SR.

### 2.6. Step 6: Calculation of the Technical Targets

For the final step of HoQ, the technical target matrix contains the technical priority *TP* and weight *W* of each functional unit. The technical priority is calculated as

(4) $$\begin{array}{c} TP_{j}=\overset{n,m}{\underset{i,j=1}{\sum }}C_{i,j}\;*\;RW_{i}, \end{array}$$

where *C*_*i*,*j*_ is the correlation value between requirement *i* and specification *j*, *RW*_*i*_ is the relative weight, and *n* and *m* represent the numbers of SR and design specifications, respectively.

The weight *W*_*j*_ is given by

(5) $$\begin{array}{c} W_{j}=\frac{TP_{j}}{\sum_{j=1}^{m}TP_{j}}, \end{array}$$

where *TP*_*j*_ is the technical priority of design specification *j* and *m* is the number of the design specifications.

## 3. Results and Discussion

### 3.1. Survey Results (Stakeholder Requirements)

The data collected from the questionnaire were analyzed using SPSS. Qualitative data were presented through numbers and percentages. Quantitative data were presented using the arithmetic mean, standard error of the mean, and standard deviation. The chi-squared test was used to test the statistical significance of the differences between qualitative data. The Kolmogorov test was done to test the linearity of quantitative variables. Parametric variables were compared between the two groups using the independent sample *t*-test. Nonparametric variables were compared between the two groups using the Mann–Whitney test.

Table 2 shows some demographic characteristics of the respondents. The majority of respondents are ED physicians, working in general hospitals, with 5 to 10 years of ED experience, and they are dealing with ED patients on a daily basis. A high percentage of respondents (72.2%) did not participate in the ED design phases before, which is an indicator of the absence of stakeholder voices during planning phases in these countries.

The responses in Table 3 showed that the most important requirement was the allocation of a suitable space for the resuscitation and trauma room, whereas the lowest one was the allocation of enough space for a library. The respondents reported that many patients admitted to the ED can be treated outside. Therefore, they rated the triage-of-initial-admission unit as highly important. The aim of such triage is to decide if patients will be treated in this ED, or they will be directed to other medical services.

### 3.2. Results of the Proposed QFD-Based ED Design Method

The QFD-based design method was applied on two EDs with the same trauma category (level-4 trauma). The first department (ED1) is located within a general hospital with 300 beds in Saudi Arabia. This department includes 17 treatment beds and receives around 125,000 patients annually. The second department (ED2) is located at a general hospital with 365 beds in Egypt. It includes 8 treatment beds and receives around 73,000 patients annually.

**Figure 3:**
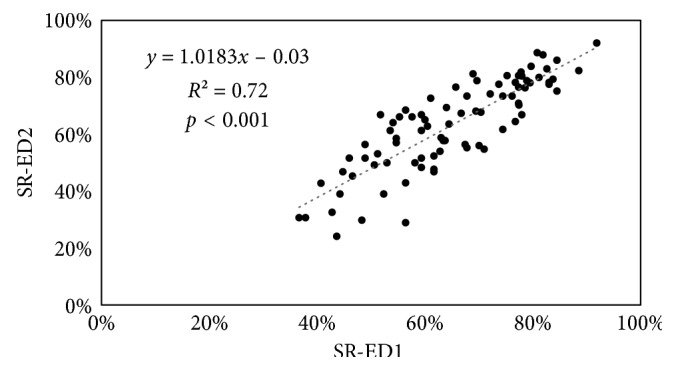
Scatter plot showing a significant correlation (*r* = 0.85) between SR of the two departments reported in this study (ED1 and ED2).

The stakeholders in both countries were asked to answer the questionnaire and mention any additional requirements that could improve the ED design and streamline the flow of an ED (with 4th trauma level). For the two EDs, the stakeholders assigned different scores for the same requirements. The statistical comparison between responses of stakeholders from both departments showed (1) a positive linear relationship between responses (*r* = 0.85) with a significant correlation (*p* < 0.001) (Figure 3), (2) nonsignificant differences in 75 requirements and significant differences in 9 requirements, and (3) no significant difference in the demographic data of respondents from both countries.

The steps of the proposed QFD-based design method (mentioned earlier in Section 2) were then applied to calculate the weights and priorities of functional units (Table 4). The results showed that the same functional unit may attain different ranks in different departments, according to the contribution of the functional unit to the mission criticality.

To validate the results, we calculated the difference ratios *R*_*u*_ between the SR and each of (1) the existing design (EXG), and (2) the QFD-based design. This difference is calculated for each functional unit as follows:

(6) $$\begin{array}{c} R_{u}=\frac{\left(A_{d,u}-A_{sr,u}\right)}{A_{sr,u}}, \end{array}$$

where *A*_*sr*,*u*_ is the area recommended by stakeholders for a given functional unit *u*, and *A*_*d, u*_ is the area according to the existing design or QFD-based design.

**Figure 4:**
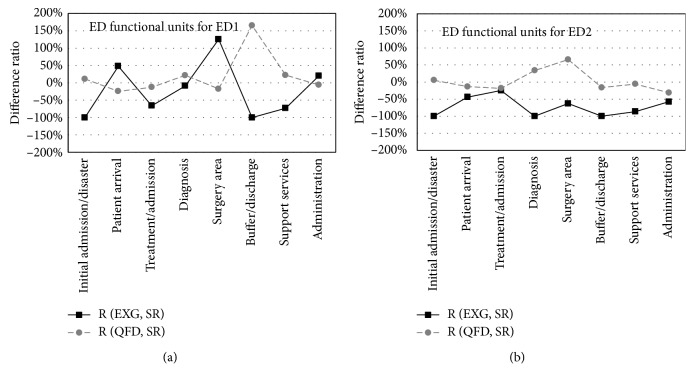
Difference ratios *R*_*u*_ for (a) ED1 and (b) ED2. The black line represents *R*_*u*_ between areas of existing design (EXG) and areas according to stakeholder requirements (SR). The dashed line represents *R*_*u*_ between areas obtained by the QFD-based design and areas according to SR.

Figure 4 shows a big discrepancy between SR and the EXG (black line) in most ED functional units for both departments. The absence of some functional units from existing designs leads to a negative 100-percent ratio. The QFD method (dashed line) showed improved difference ratios for both departments.

In general, the results showed a significant linear relationship between the results obtained by QFD-based design method and the stakeholder needs (*r* = 0.92 for ED1 and *r* = 0.93 for ED2). In spite of this linear relation, some units obtained different weights compared to the stakeholder evaluation to satisfy the IS. The results of the QFD-based design method showed a positive correlation to the IS (*r* = 0.94 for ED1 and *r* = 0.91 for ED2). Design and planning teams can use the resulting weights and ranks to allocate, increase, and decrease areas of some functional units to satisfy the stakeholders, streamline the flow within the ED, and to alleviate the crowding problem.

## 4. Conclusion

The QFD method was developed to assign priorities and weights for the ED functional units. If spaces or budgets are not enough to cover all units of ED, the medical planners can use this prioritization scheme for paying the appropriate attention to each unit based on its weight. The main goal of this work was to reassess the major design solutions for ED that would improve the flow within the ED based on both the SR and IS. The adaptation and implementation of the QFD method helped in introducing a simple and realistic concept design that could achieve that goal. This is due to the ability of QFD to translate the stakeholder needs into design requirements.

The main challenge of the proposed method was the reliance on experts to develop the relationship matrix. Our framework is flexible to be used for any ED, and it can generate variable weights and priorities for the same units over time. As we did not identify the subunits (components) of each functional unit in this work, the medical planners can identify these subunits based on the operational model, types of patient conditions, and the location of each hospital. A separate future work shall use the second phase of QFD to focus on the identification and prioritization of the subunits to propose a complete list of ED services.

## Figures and Tables

**Table 1:** Design specifications for QFD-based ED design and their functions.

Design specification	Function
Initial admission and disaster management	Register and sort all patients coming to ED to decide if they will be treated in this ED, or will be directed to other medical services. This zone must be disaster-ready.
Patient arrival	Receive, register, identify the level of urgency, and handle ED patients upon arrival.
Patient treatment and admission	Provide care for unstable and critical patients, and low-acuity patients who stay for more than 2 hours.
Diagnostic unit	Radiology and laboratory tests.
Surgical unit	Plastering, performing minor procedures, and patient recovery.
Support services	Equipment storage, dirty and utility rooms, general stores, etc.
Administrative unit	Staff offices, stations, change rooms, lounge, seminar room, and library if needed.
Temporary buffer and discharge	Hosting patients who need less treatment, or are waiting for transportation, or are going to be admitted to inpatient care.

**Table 2:** Some of the demographic characteristics of respondents.

Demographic data of respondents (stakeholders)	Valid sample (*N*=108)	
	No.	%
*Country they work in*		
KSA	59	54.6
Egypt	49	45.4

*Profession*		
Clinical engineer	25	23.1
Medical planner	20	18.5
Nurse/pharmacist	12	11.1
ED physician	39	36.1
Non-ED physician	12	11.1

*Years of experience within ED*		
None	14	13.0
<5	34	31.5
5–10	44	40.7
10–15	9	8.3
15+	7	6.5

*Frequency of interaction with patients in the ED*		
Seldom	31	28.7
On consultation	31	28.7
Daily	46	42.6

*Participation in ED design*		
No	78	72.2
Yes	30	27.8

**Table 3:** A sample of survey responses showing the mean importance of some ED requirements.

No	Stakeholder requirements	Mean importance (%)	Std. error of mean	Std. deviation
1	Trauma and resuscitation room	91.43	1.81	17.93
2	Triage in the initial admission unit	84.49	1.96	19.38
3	Wounded patients holding area	65.06	3.03	27.59
4	Ambulance entrance store	64.37	3.36	31.35
5	Dental room	36.14	3.33	30.31
6	Kids playing area (playground)	34.34	3.31	30.17
7	Library	32.93	3.36	30.40

**Table 4:** Priorities and relative weights of design specifications (functional units) according to the QFD-based design method for the two emergency departments reported in this work (ED1 and ED2).

Functional units	ED1		ED2	
	Relative weight (%)	Rank	Relative weight (%)	Rank
Patient treatment and admission	27.1	1	15.1	3
Patient arrival	21.3	2	28.6	1
Temporary buffer and discharge	13.7	3	5.9	6
Diagnostic unit	12.2	4	18.1	2
Administrative unit	11.4	5	11.3	4
Initial admission and disaster management	5.6	6	5.6	7
Support services	5.0	7	5.2	8
Surgical unit	3.7	8	10.1	5

## Data Availability

The data used to support the findings of this study are available from the corresponding author upon request.
