# Corrigendum to “Functional and Spatial Design of Emergency Departments Using Quality Function Deployment”

**DOI:** 10.1155/2019/8240684

**Published:** 2019-07-17

**Authors:** Yassin Abdelsamad, Muhammad Rushdi, Bassel Tawfik

**Affiliations:** Department of Biomedical Engineering and Systems, Faculty of Engineering, Cairo University, Giza 12613, Egypt

In the article titled “Functional and Spatial Design of Emergency Departments Using Quality Function Deployment” [1], there was an error in equation (4). Although this error does not affect the results or the conclusions, the double indexes *i*, *j* and *n*, *m* might be confusing for the reader. Therefore, equation (4) should be corrected as follows.

For the final step of HoQ, the technical target matrix contains the technical priority TP and the relative weight RW of each functional unit. The technical priority is calculated as

(4) $$\begin{array}{c} {\text{TP}}_{j}=\overset{n}{\underset{i=1}{\sum }}C_{i,j}\;*\;{\text{RW}}_{i}, \end{array}$$

where *C*_*i,j*_ is the correlation value between requirement *i* and specification *j*, RW_*i*_ is the relative weight, and *n* represents the number of SRs.
